# Death of Dr. John Houston

**Published:** 1845-10

**Authors:** 


We have to record the death of one of our oldest contributors, Dr. John Houston, M. R. I. A., ex-Curator to the Museum of the Royal College of Surgeons, and one of the Lecturers on Surgery in the School of Medicine, Park-street, which took place since the publication of our last Number. This talented member of the medical profession had laboured for many years, and not in vain, to advance the cause of zoological and anatomical science, and practical surgery, pathology, and general medical literature in Ireland. The recent date of the event precludes the possibility of a more extended notice; but we hope, in a future number, to bring a short summary of his writings before our readers.—*Dublin Journal.*

